# Antimicrobial resistance pattern of gram negative bacteria to 3rd and 4th generation cephalosporins

**DOI:** 10.1186/1753-6561-5-S6-P146

**Published:** 2011-06-29

**Authors:** P Revathi, T Jeyaseelan Senthinath, R Vigneswari

**Affiliations:** 1 Pharmacology, Chennai Medical College Hospital and Research Centre, Trichy, India; 2 Microbiology, Chennai Medical College Hospital and Research Centre, Trichy, India

## Introduction / objectives

Antimicrobial resistance (AMR) is increasing & the status in rural areas is less known. To find out the AMR pattern of clinical isolates of selected gram negative bacilli (GNB) to 3^rd^ and 4^th^generation cephalosporins (GCPs) at a rural and urban tertiary care hospital.

## Methods

A total 2366(Rural= 1016; Urban=1350) isolates (various clinical samples) of GNB collected over 3 consecutive months were studied for AMR pattern of selected 3^rd^ and 4^th^ GCPs. The data were entered in WHONET & analysed& compared with each other.

## Results

In rural & urban isolates the AMR pattern for 3^rd^ / 4^th^GCPs varied from 60-100 /32-100 &34-50/21-58 respectively to selected GNB, & it was significantly more among rural isolates. Among GNB, AMR was low with E.Coli & Klebsiella but high among Pseudomonas.

## Conclusion

A heterogeneous AMR pattern observed in the study was comparable with published reports. The probable reasons for wide variations were due to overuse, misuse& dysregulated availability of antimicrobials; promotional incentives & high profit margins; & demands of the community. These push the rural practitioners to use newer generation of antimicrobials frequently. Also lack of adherence to medication, suboptimal regulatory system (Antibiotic policy, Monitoring), lack of prescribing policies and to some extent technical aspects of GNB, might have contributed to increased AMR in rural areas. Hence there is an urgent need to design & implement antimicrobial policy & surveillance system at regional & institutional level under national guidelines.

## Disclosure of interest

None declared.

